# Crystal structure of ethyl 4-(2-fluoro­phen­yl)-6-methyl-2-sulfanyl­idene-1,2,3,4-tetra­hydro­pyrimidine-5-carboxyl­ate

**DOI:** 10.1107/S2056989015015145

**Published:** 2015-09-12

**Authors:** M. S. Krishnamurthy, Noor Shahina Begum

**Affiliations:** a Department of Studies in Chemistry, Central College Campus, Bangalore University, Bangalore 560 001, Karnataka, India

**Keywords:** crystal structure, ester, pyrimidine, hydrogen bonding, 3,4-di­hydro­pyrimidin-2(1*H*)-one, therapeutic properties, pharmacological properties

## Abstract

The title compound, C_14_H_15_FN_2_O_2_S, crystallizes with two mol­ecules in the asymmetric unit. In each mol­ecule, the pyrimidine ring adopts a sofa conformation with the *sp* ^3^-hybridized C atom forming the flap and the fluoro-substituted ring in an axial position. In the crystal, mol­ecules are linked *via* N—H⋯S hydrogen bonds, forming chains of *R*_2_^2^(8) rings along [100]. In one independent mol­ecule, an intra­molecular C—H⋯O hydrogen bond is observed.

## Related literature   

For the therapeutic and pharmacological properties of 3,4-di­hydro­pyrimidin-2(1*H*)-ones, see: Kappe (2000 ▸); Hurst & Hull (1961 ▸); Mayer *et al.* (1999 ▸); Atwal *et al.* (1991 ▸). For their applications in calcium-channel modulators, see: Kappe (1998 ▸); Jauk *et al.* (2000 ▸); Krishnamurthy & Begum (2015 ▸). For the bioactivity of organo–fluorine compounds, see: Hermann *et al.* (2003 ▸); Ulrich (2004 ▸). For examples of fluorine-directed crystal packing, see: Prasanna & Guru Row (2001 ▸). For related structures, see: Qin *et al.* (2006 ▸); Krishnamurthy & Begum (2015 ▸). For hydrogen-bond graph-set notation, see: Bernstein *et al.* (1995 ▸).
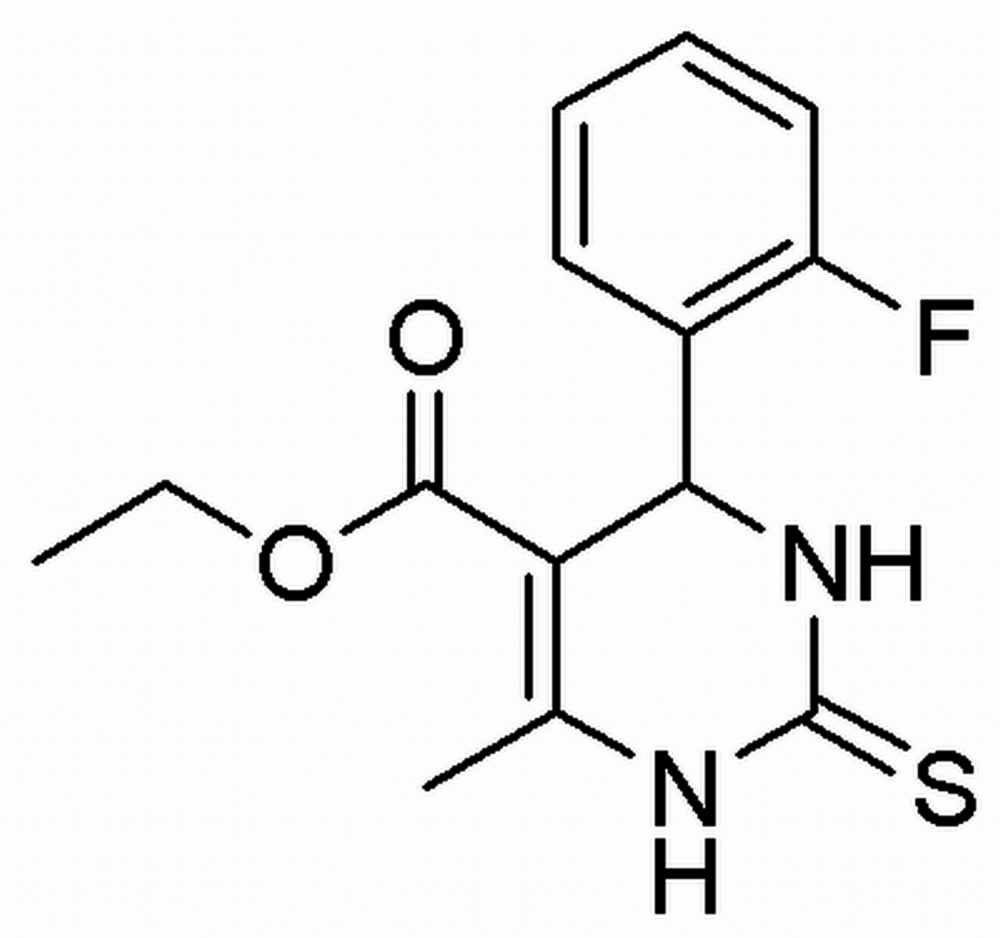


## Experimental   

### Crystal data  

C_14_H_15_FN_2_O_2_S
*M* *_r_* = 294.34
Triclinic, 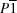

*a* = 8.9298 (6) Å
*b* = 11.5870 (8) Å
*c* = 15.7459 (11) Å
α = 100.940 (2)°
β = 104.804 (2)°
γ = 98.153 (2)°
*V* = 1515.11 (18) Å^3^
*Z* = 4
Mo *K*α radiation
μ = 0.23 mm^−1^
*T* = 100 K
0.18 × 0.16 × 0.16 mm

### Data collection  

Bruker SMART APEX CCD diffractometer
Absorption correction: multi-scan (*SADABS*; Bruker, 1998 ▸) *T*_min_ = 0.960, *T*_max_ = 0.965
17808 measured reflections
5328 independent reflections
3389 reflections with *I* > 2σ(*I*)
*R*_int_ = 0.039

### Refinement  

*R*[*F* ^2^ > 2σ(*F* ^2^)] = 0.075
*wR*(*F* ^2^) = 0.228
*S* = 0.98
5328 reflections
365 parameters
H-atom parameters constrained
Δρ_max_ = 1.04 e Å^−3^
Δρ_min_ = −0.36 e Å^−3^

### 

Data collection: *SMART* (Bruker, 1998 ▸); cell refinement: *SMART*; data reduction: *SAINT-Plus* (Bruker, 1998 ▸); program(s) used to solve structure: *SHELXS97* (Sheldrick, 2008 ▸); program(s) used to refine structure: *SHELXL97* (Sheldrick, 2008 ▸); molecular graphics: *ORTEP-3 for Windows* (Farrugia, 2012 ▸), *CAMERON* (Watkin *et al.*, 1996 ▸) and *DIAMOND* (Brandenburg & Berndt, 1999 ▸); software used to prepare material for publication: *WinGX* (Farrugia, 2012 ▸).

## Supplementary Material

Crystal structure: contains datablock(s) global, I. DOI: 10.1107/S2056989015015145/lh5781sup1.cif

Structure factors: contains datablock(s) I. DOI: 10.1107/S2056989015015145/lh5781Isup2.hkl

Supporting information file. DOI: 10.1107/S2056989015015145/lh5781Isup3.cml

**Fig. 1.**
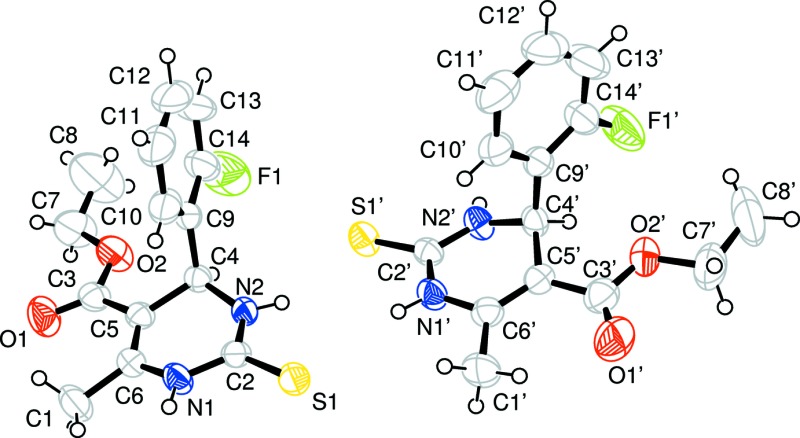
The asymmetric unit of the title compound with displacement ellipsoids drawn at the 50% probability level. H atoms are presented as small spheres of arbitrary radius.

. DOI: 10.1107/S2056989015015145/lh5781fig1.tif
The asymmetric unit of the title compound with displacement ellipsoids drawn at the 50% probability level. H atoms are presented as small spheres of arbitrary radius.

**Fig. 2.**
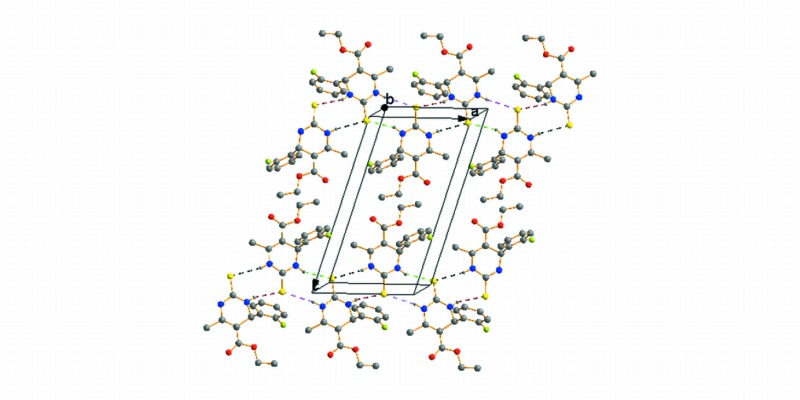
Part of the crystal structure with hydrogen bonds shown as dashed lines.

. DOI: 10.1107/S2056989015015145/lh5781fig2.tif
Part of the crystal structure with hydrogen bonds shown as dashed lines.

CCDC reference: 1418668

Additional supporting information:  crystallographic information; 3D view; checkCIF report

## Figures and Tables

**Table 1:** Hydrogen-bond geometry (, )

*D*H*A*	*D*H	H*A*	*D* *A*	*D*H*A*
N1H1S1^i^	0.88	2.83	3.703(4)	170
N1H1S1	0.88	2.84	3.711(4)	171
N2H2S1	0.88	2.52	3.337(4)	155
N2H2S1^ii^	0.88	2.50	3.335(5)	158
C1H11O1	0.98	2.14	2.861(6)	129

Symmetry codes: (i) 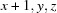; (ii) 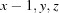.

## supplementary crystallographic information

### S1. Comment

The Biginelli reaction is a three-component condensation of ethyl acetoacetate, benzaldehyde and urea for the synthesis of 3,4-dihydropyrimidine-2(*1H*) -ones (abbreviated as DHPMs). DHPMs have recently emerged as important target molecules because of their therapeutic and pharmacological properties (Kappe, 2000), such as antiviral (Hurst & Hull, 1961), antimitotic (Mayer *et al.*, 1999), anticarcinogenic and antihypertensive (Atwal *et al.*, 1991). They are also noteworthy as calcium channel modulators (Kappe, 1998; Jauk *et al.*, 2000). In addition, compounds that contain fluorine have special bioactivity, *e.g.* flumioxazin is a widely used herbicide (Hermann *et al.*, 2003; Ulrich, 2004). Guru Row and co-workers have extensively studied the structural property of fluorine and they have presented several elegant examples of fluorine directed crystal packing (Prasanna & Guru Row, 2001). Herein, we report the crystal structure of the title compound.

The asymmetric unit of the title compound is shown in Fig. 1. There are two indpendent molecules in the asymmetric unit. The bond lengths and bond angles are in good agreement with the corresponding bond distances and angles reported in closely related structures (Qin *et al.*, 2006; Krishnamurthy & Begum, 2015). In each molecule, the pyrimidine ring adopts a *sofa* conformation with the sp^3^ hybridized carbon atom [C4 and C4'] forming the flap and the fluoro-substituted ring in an axial position. The carbonyl group of the exocyclic ester at C5 and C5' adopts a *cis* orientation with respect to the C5═C6 and C5'═C6 double bond. The fluoro-substituted benzene ring adopts an *syn* periplanar conformation with respect to the C4—H4 and C4'—H4' bonds. In the crystal, molecules are linked *via* N—H···S hydrogen bonds forming chains of *R*_2_^2^(8) rings (Bernstein *et al.*, 1995) along [100] (Fig. 2). In one independent molecule an intramolecular C—H···O hydrogen bond is observed.

### S2. Experimental

The title compound was synthesized by the reaction of 2-fluorobenzaldehyde (1.24 g, 10 mmol), ethylacetoacetate (1.52 g, 12 mmol) and thiourea (1.14 g, 15 mmol) in 15 ml ethanol was refluxed for 6 h in the presence of concentrated hydrochloric acid as a catalyst. The reaction was monitored with TLC and the reaction medium was quenched in ice cold water. The precipitate obtained was filtered and dried. The compound was recrystallized from ethanol solvent by slow evaporation method, yielding colorless blocks suitable for X-ray diffraction studies (yield 76%; m.p. 485 K).

### S3. Refinement

The H atoms were placed in calculated positions in a riding-model approximation with N—H = 0.86° A; C—H = 0.93° A, 0.96 ° A and 0.97 ° A for aromatic, methyl and methylene H-atoms respectively, with *U*_iso_(H) = 1.5*U*_eq_(C) for methyl H atoms and *U*_iso_(H) = 1.2U_eq_(C) for other hydrogen atoms.

### Crystal data

**Table:**

C_14_H_15_FN_2_O_2_S	*Z* = 4
*M**_r_* = 294.34	*F*(000) = 616
Triclinic, *P*1	*D*_x_ = 1.290 Mg m^−^^3^
Hall symbol: -P 1	Mo *K*α radiation, λ = 0.71073 Å
*a* = 8.9298 (6) Å	Cell parameters from 5328 reflections
*b* = 11.5870 (8) Å	θ = 2.4–25.0°
*c* = 15.7459 (11) Å	µ = 0.23 mm^−^^1^
α = 100.940 (2)°	*T* = 100 K
β = 104.804 (2)°	Block, colourless
γ = 98.153 (2)°	0.18 × 0.16 × 0.16 mm
*V* = 1515.11 (18) Å^3^	

### Data collection

**Table:**

Bruker SMART APEX CCD diffractometer	5328 independent reflections
Radiation source: fine-focus sealed tube	3389 reflections with *I* > 2σ(*I*)
Graphite monochromator	*R*_int_ = 0.039
ω scans	θ_max_ = 25.0°, θ_min_ = 2.4°
Absorption correction: multi-scan (*SADABS*; Bruker, 1998)	*h* = −10→10
*T*_min_ = 0.960, *T*_max_ = 0.965	*k* = −13→13
17808 measured reflections	*l* = −18→18

### Refinement

**Table:**

Refinement on *F*^2^	Primary atom site location: structure-invariant direct methods
Least-squares matrix: full	Secondary atom site location: difference Fourier map
*R*[*F*^2^ > 2σ(*F*^2^)] = 0.075	Hydrogen site location: inferred from neighbouring sites
*wR*(*F*^2^) = 0.228	H-atom parameters constrained
*S* = 0.98	*w* = 1/[σ^2^(*F*_o_^2^) + (0.1269*P*)^2^ + 1.4728*P*] where *P* = (*F*_o_^2^ + 2*F*_c_^2^)/3
5328 reflections	(Δ/σ)_max_ = 0.001
365 parameters	Δρ_max_ = 1.04 e Å^−^^3^
0 restraints	Δρ_min_ = −0.35 e Å^−^^3^

### Special details

**Table:**

Geometry. All s.u.'s (except the s.u. in the dihedral angle between two l.s. planes) are estimated using the full covariance matrix. The cell s.u.'s are taken into account individually in the estimation of s.u.'s in distances, angles and torsion angles; correlations between s.u.'s in cell parameters are only used when they are defined by crystal symmetry. An approximate (isotropic) treatment of cell s.u.'s is used for estimating s.u.'s involving l.s. planes.
Refinement. Refinement of F^2^ against ALL reflections. The weighted R-factor wR and goodness of fit S are based on F^2^, conventional R-factors R are based on F, with F set to zero for negative F^2^. The threshold expression of F^2^ > 2σ(F^2^) is used only for calculating R-factors(gt) etc. and is not relevant to the choice of reflections for refinement. R-factors based on F^2^ are statistically about twice as large as those based on F, and R- factors based on ALL data will be even larger.

### Fractional atomic coordinates and isotropic or equivalent isotropic displacement parameters (Å^2^)

**Table:**

	*x*	*y*	*z*	*U*_iso_*/*U*_eq_	
S1	0.86288 (13)	0.90033 (10)	0.07601 (7)	0.0522 (3)	
O1	0.6733 (4)	0.6314 (3)	−0.3693 (2)	0.0743 (10)	
O2	0.4450 (4)	0.6240 (3)	−0.33627 (19)	0.0701 (10)	
N1	0.8792 (4)	0.8058 (3)	−0.0880 (2)	0.0489 (9)	
H1	0.9822	0.8229	−0.0631	0.059*	
N2	0.6330 (4)	0.7993 (3)	−0.0734 (2)	0.0414 (8)	
H2	0.5717	0.8312	−0.0439	0.050*	
F1	0.2317 (4)	0.6093 (4)	−0.2084 (3)	0.1226 (14)	
C1	0.9490 (6)	0.7543 (5)	−0.2257 (3)	0.0738 (15)	
H1A	0.9492	0.6715	−0.2544	0.111*	
H1B	1.0519	0.7900	−0.1810	0.111*	
H1C	0.9294	0.8010	−0.2717	0.111*	
C2	0.7856 (5)	0.8314 (3)	−0.0339 (3)	0.0412 (9)	
C3	0.6001 (5)	0.6538 (4)	−0.3149 (3)	0.0499 (10)	
C4	0.5536 (4)	0.7162 (3)	−0.1611 (2)	0.0382 (9)	
H4	0.4611	0.7476	−0.1922	0.046*	
C5	0.6672 (5)	0.7122 (3)	−0.2183 (3)	0.0415 (9)	
C6	0.8224 (5)	0.7548 (4)	−0.1798 (3)	0.0460 (10)	
C7	0.3610 (7)	0.5580 (6)	−0.4281 (3)	0.0891 (19)	
H7A	0.3593	0.6128	−0.4694	0.107*	
H7B	0.4162	0.4939	−0.4471	0.107*	
C8	0.2083 (10)	0.5082 (10)	−0.4334 (5)	0.172 (5)	
H8A	0.2091	0.4715	−0.3822	0.258*	
H8B	0.1620	0.4467	−0.4901	0.258*	
H8C	0.1453	0.5708	−0.4319	0.258*	
C9	0.4933 (5)	0.5941 (4)	−0.1489 (3)	0.0455 (10)	
C10	0.5973 (7)	0.5272 (4)	−0.1092 (3)	0.0641 (13)	
H10	0.7080	0.5584	−0.0905	0.077*	
C11	0.5406 (10)	0.4154 (5)	−0.0968 (4)	0.091 (2)	
H11	0.6121	0.3698	−0.0706	0.109*	
C12	0.3822 (15)	0.3718 (6)	−0.1225 (5)	0.121 (3)	
H12	0.3439	0.2959	−0.1130	0.145*	
C13	0.2769 (9)	0.4347 (7)	−0.1617 (5)	0.103 (2)	
H13	0.1664	0.4031	−0.1807	0.123*	
C14	0.3357 (6)	0.5445 (5)	−0.1726 (3)	0.0669 (13)	
S1'	0.31495 (13)	0.84530 (11)	−0.00790 (7)	0.0544 (4)	
O1'	0.6197 (5)	1.1437 (4)	0.4307 (3)	0.0940 (13)	
O2'	0.3597 (4)	1.0906 (3)	0.4089 (2)	0.0683 (9)	
N1'	0.5140 (4)	0.9516 (3)	0.1541 (2)	0.0490 (9)	
H1'	0.5889	0.9363	0.1291	0.059*	
N2'	0.2541 (4)	0.9254 (3)	0.1455 (2)	0.0463 (8)	
H2'	0.1559	0.9156	0.1118	0.056*	
F1'	0.0344 (4)	0.9094 (4)	0.3206 (3)	0.1074 (12)	
C1'	0.7322 (5)	1.0702 (5)	0.2784 (3)	0.0692 (14)	
H1'1	0.7651	1.0866	0.3446	0.104*	
H1'2	0.7528	1.1453	0.2595	0.104*	
H1'3	0.7921	1.0141	0.2544	0.104*	
C2'	0.3619 (5)	0.9103 (3)	0.1028 (3)	0.0428 (9)	
C3'	0.4889 (6)	1.0926 (4)	0.3814 (3)	0.0603 (12)	
C4'	0.2823 (5)	0.9565 (4)	0.2428 (3)	0.0448 (10)	
H4'	0.2094	1.0105	0.2567	0.054*	
C5'	0.4504 (5)	1.0246 (3)	0.2879 (3)	0.0454 (10)	
C6'	0.5595 (5)	1.0163 (4)	0.2430 (3)	0.0475 (10)	
C7'	0.3799 (8)	1.1522 (5)	0.5011 (4)	0.0890 (18)	
H7'1	0.4179	1.2393	0.5096	0.107*	
H7'2	0.4591	1.1219	0.5431	0.107*	
C8'	0.2281 (11)	1.1309 (9)	0.5198 (5)	0.144 (3)	
H8'1	0.1539	1.1693	0.4828	0.217*	
H8'2	0.2423	1.1647	0.5839	0.217*	
H8'3	0.1862	1.0443	0.5052	0.217*	
C9'	0.2458 (5)	0.8451 (4)	0.2768 (3)	0.0467 (10)	
C10'	0.3336 (6)	0.7567 (4)	0.2711 (3)	0.0623 (13)	
H10'	0.4194	0.7666	0.2460	0.075*	
C11'	0.2998 (8)	0.6546 (5)	0.3007 (4)	0.0861 (18)	
H11'	0.3602	0.5939	0.2946	0.103*	
C12'	0.1789 (9)	0.6404 (6)	0.3392 (4)	0.091 (2)	
H12'	0.1561	0.5704	0.3604	0.109*	
C13'	0.0928 (8)	0.7260 (7)	0.3468 (4)	0.0926 (19)	
H13'	0.0103	0.7176	0.3745	0.111*	
C14'	0.1248 (6)	0.8256 (5)	0.3142 (3)	0.0675 (13)	

### Atomic displacement parameters (Å^2^)

**Table:**

	*U*^11^	*U*^22^	*U*^33^	*U*^12^	*U*^13^	*U*^23^
S1	0.0422 (6)	0.0611 (7)	0.0453 (6)	0.0011 (5)	0.0124 (5)	0.0006 (5)
O1	0.076 (2)	0.100 (3)	0.0456 (18)	0.012 (2)	0.0265 (18)	0.0051 (17)
O2	0.053 (2)	0.103 (3)	0.0393 (17)	−0.0082 (18)	0.0078 (14)	0.0048 (16)
N1	0.0334 (18)	0.065 (2)	0.044 (2)	0.0023 (16)	0.0144 (15)	0.0047 (17)
N2	0.0351 (18)	0.0450 (18)	0.0419 (18)	0.0053 (15)	0.0147 (15)	0.0024 (15)
F1	0.057 (2)	0.161 (4)	0.132 (3)	−0.004 (2)	0.017 (2)	0.025 (3)
C1	0.057 (3)	0.099 (4)	0.063 (3)	−0.001 (3)	0.034 (3)	0.003 (3)
C2	0.039 (2)	0.037 (2)	0.047 (2)	0.0044 (17)	0.0133 (19)	0.0094 (18)
C3	0.057 (3)	0.049 (2)	0.045 (2)	0.005 (2)	0.016 (2)	0.017 (2)
C4	0.034 (2)	0.041 (2)	0.039 (2)	0.0074 (17)	0.0101 (17)	0.0083 (17)
C5	0.048 (2)	0.040 (2)	0.038 (2)	0.0046 (18)	0.0154 (18)	0.0136 (17)
C6	0.047 (2)	0.046 (2)	0.046 (2)	0.0057 (19)	0.020 (2)	0.0094 (19)
C7	0.076 (4)	0.117 (5)	0.044 (3)	−0.023 (3)	0.001 (3)	0.001 (3)
C8	0.102 (6)	0.268 (12)	0.072 (5)	−0.073 (7)	0.008 (4)	−0.030 (6)
C9	0.052 (2)	0.045 (2)	0.037 (2)	0.000 (2)	0.0194 (19)	0.0025 (18)
C10	0.097 (4)	0.047 (3)	0.059 (3)	0.016 (3)	0.037 (3)	0.016 (2)
C11	0.158 (7)	0.054 (3)	0.086 (4)	0.026 (4)	0.068 (4)	0.027 (3)
C12	0.216 (10)	0.057 (4)	0.095 (5)	−0.029 (5)	0.098 (6)	0.001 (4)
C13	0.107 (5)	0.087 (5)	0.096 (5)	−0.045 (4)	0.051 (4)	−0.003 (4)
C14	0.055 (3)	0.075 (3)	0.063 (3)	−0.008 (3)	0.022 (3)	0.006 (3)
S1'	0.0444 (6)	0.0736 (8)	0.0449 (6)	0.0117 (5)	0.0137 (5)	0.0122 (5)
O1'	0.076 (3)	0.107 (3)	0.067 (2)	−0.007 (2)	0.008 (2)	−0.017 (2)
O2'	0.077 (2)	0.071 (2)	0.0534 (19)	0.0092 (18)	0.0289 (17)	−0.0019 (16)
N1'	0.0341 (18)	0.062 (2)	0.049 (2)	0.0068 (16)	0.0149 (16)	0.0067 (17)
N2'	0.0359 (18)	0.066 (2)	0.0409 (19)	0.0151 (16)	0.0118 (15)	0.0167 (16)
F1'	0.083 (2)	0.135 (3)	0.138 (3)	0.034 (2)	0.074 (2)	0.047 (2)
C1'	0.042 (3)	0.084 (3)	0.070 (3)	−0.003 (2)	0.014 (2)	0.005 (3)
C2'	0.038 (2)	0.045 (2)	0.050 (2)	0.0109 (18)	0.0160 (19)	0.0165 (19)
C3'	0.069 (3)	0.050 (3)	0.060 (3)	0.010 (2)	0.018 (3)	0.011 (2)
C4'	0.041 (2)	0.051 (2)	0.046 (2)	0.0133 (19)	0.0185 (19)	0.0086 (19)
C5'	0.048 (2)	0.042 (2)	0.045 (2)	0.0080 (19)	0.012 (2)	0.0090 (18)
C6'	0.042 (2)	0.046 (2)	0.051 (3)	0.0055 (19)	0.012 (2)	0.010 (2)
C7'	0.134 (6)	0.077 (4)	0.057 (3)	0.021 (4)	0.039 (4)	0.004 (3)
C8'	0.153 (7)	0.189 (8)	0.105 (6)	0.029 (6)	0.092 (6)	−0.004 (5)
C9'	0.045 (2)	0.052 (2)	0.038 (2)	0.003 (2)	0.0100 (19)	0.0067 (19)
C10'	0.082 (3)	0.049 (3)	0.057 (3)	0.007 (2)	0.025 (3)	0.012 (2)
C11'	0.126 (5)	0.054 (3)	0.069 (4)	0.016 (3)	0.014 (4)	0.013 (3)
C12'	0.121 (5)	0.074 (4)	0.065 (4)	−0.022 (4)	0.022 (4)	0.024 (3)
C13'	0.094 (5)	0.102 (5)	0.083 (4)	−0.014 (4)	0.040 (4)	0.031 (4)
C14'	0.061 (3)	0.076 (3)	0.064 (3)	0.000 (3)	0.027 (3)	0.012 (3)

### Geometric parameters (Å, º)

**Table:**

S1—C2	1.679 (4)	S1'—C2'	1.679 (4)
O1—C3	1.216 (5)	O1'—C3'	1.218 (6)
O2—C3	1.316 (5)	O2'—C3'	1.330 (6)
O2—C7	1.450 (5)	O2'—C7'	1.443 (6)
N1—C2	1.360 (5)	N1'—C2'	1.354 (5)
N1—C6	1.382 (5)	N1'—C6'	1.383 (5)
N1—H1	0.8800	N1'—H1'	0.8800
N2—C2	1.311 (5)	N2'—C2'	1.321 (5)
N2—C4	1.463 (5)	N2'—C4'	1.452 (5)
N2—H2	0.8800	N2'—H2'	0.8800
F1—C14	1.355 (6)	F1'—C14'	1.354 (6)
C1—C6	1.490 (6)	C1'—C6'	1.496 (6)
C1—H1A	0.9800	C1'—H1'1	0.9800
C1—H1B	0.9800	C1'—H1'2	0.9800
C1—H1C	0.9800	C1'—H1'3	0.9800
C3—C5	1.469 (6)	C3'—C5'	1.459 (6)
C4—C9	1.506 (5)	C4'—C5'	1.512 (6)
C4—C5	1.518 (5)	C4'—C9'	1.513 (6)
C4—H4	1.0000	C4'—H4'	1.0000
C5—C6	1.340 (6)	C5'—C6'	1.346 (6)
C7—C8	1.378 (9)	C7'—C8'	1.456 (9)
C7—H7A	0.9900	C7'—H7'1	0.9900
C7—H7B	0.9900	C7'—H7'2	0.9900
C8—H8A	0.9800	C8'—H8'1	0.9800
C8—H8B	0.9800	C8'—H8'2	0.9800
C8—H8C	0.9800	C8'—H8'3	0.9800
C9—C14	1.368 (6)	C9'—C14'	1.368 (6)
C9—C10	1.394 (6)	C9'—C10'	1.378 (6)
C10—C11	1.391 (7)	C10'—C11'	1.375 (7)
C10—H10	0.9500	C10'—H10'	0.9500
C11—C12	1.359 (11)	C11'—C12'	1.371 (9)
C11—H11	0.9500	C11'—H11'	0.9500
C12—C13	1.366 (11)	C12'—C13'	1.345 (9)
C12—H12	0.9500	C12'—H12'	0.9500
C13—C14	1.365 (8)	C13'—C14'	1.373 (8)
C13—H13	0.9500	C13'—H13'	0.9500
			
C3—O2—C7	117.6 (4)	C3'—O2'—C7'	117.2 (4)
C2—N1—C6	124.0 (3)	C2'—N1'—C6'	124.3 (3)
C2—N1—H1	118.0	C2'—N1'—H1'	117.8
C6—N1—H1	118.0	C6'—N1'—H1'	117.8
C2—N2—C4	127.1 (3)	C2'—N2'—C4'	126.1 (3)
C2—N2—H2	116.5	C2'—N2'—H2'	117.0
C4—N2—H2	116.5	C4'—N2'—H2'	117.0
C6—C1—H1A	109.5	C6'—C1'—H1'1	109.5
C6—C1—H1B	109.5	C6'—C1'—H1'2	109.5
H1A—C1—H1B	109.5	H1'1—C1'—H1'2	109.5
C6—C1—H1C	109.5	C6'—C1'—H1'3	109.5
H1A—C1—H1C	109.5	H1'1—C1'—H1'3	109.5
H1B—C1—H1C	109.5	H1'2—C1'—H1'3	109.5
N2—C2—N1	115.6 (3)	N2'—C2'—N1'	115.6 (4)
N2—C2—S1	122.9 (3)	N2'—C2'—S1'	122.6 (3)
N1—C2—S1	121.5 (3)	N1'—C2'—S1'	121.8 (3)
O1—C3—O2	122.7 (4)	O1'—C3'—O2'	122.5 (5)
O1—C3—C5	126.7 (4)	O1'—C3'—C5'	126.7 (5)
O2—C3—C5	110.6 (4)	O2'—C3'—C5'	110.8 (4)
N2—C4—C9	110.5 (3)	N2'—C4'—C5'	109.6 (3)
N2—C4—C5	108.9 (3)	N2'—C4'—C9'	110.5 (3)
C9—C4—C5	112.3 (3)	C5'—C4'—C9'	112.3 (3)
N2—C4—H4	108.3	N2'—C4'—H4'	108.1
C9—C4—H4	108.3	C5'—C4'—H4'	108.1
C5—C4—H4	108.3	C9'—C4'—H4'	108.1
C6—C5—C3	122.6 (4)	C6'—C5'—C3'	122.6 (4)
C6—C5—C4	120.3 (3)	C6'—C5'—C4'	119.7 (4)
C3—C5—C4	117.0 (3)	C3'—C5'—C4'	117.7 (4)
C5—C6—N1	120.0 (4)	C5'—C6'—N1'	119.4 (4)
C5—C6—C1	126.9 (4)	C5'—C6'—C1'	127.2 (4)
N1—C6—C1	113.1 (4)	N1'—C6'—C1'	113.4 (4)
C8—C7—O2	110.1 (5)	O2'—C7'—C8'	108.5 (6)
C8—C7—H7A	109.6	O2'—C7'—H7'1	110.0
O2—C7—H7A	109.6	C8'—C7'—H7'1	110.0
C8—C7—H7B	109.6	O2'—C7'—H7'2	110.0
O2—C7—H7B	109.6	C8'—C7'—H7'2	110.0
H7A—C7—H7B	108.2	H7'1—C7'—H7'2	108.4
C7—C8—H8A	109.5	C7'—C8'—H8'1	109.5
C7—C8—H8B	109.5	C7'—C8'—H8'2	109.5
H8A—C8—H8B	109.5	H8'1—C8'—H8'2	109.5
C7—C8—H8C	109.5	C7'—C8'—H8'3	109.5
H8A—C8—H8C	109.5	H8'1—C8'—H8'3	109.5
H8B—C8—H8C	109.5	H8'2—C8'—H8'3	109.5
C14—C9—C10	116.4 (4)	C14'—C9'—C10'	116.2 (4)
C14—C9—C4	122.6 (4)	C14'—C9'—C4'	122.6 (4)
C10—C9—C4	120.9 (4)	C10'—C9'—C4'	121.2 (4)
C11—C10—C9	120.6 (6)	C11'—C10'—C9'	121.6 (5)
C11—C10—H10	119.7	C11'—C10'—H10'	119.2
C9—C10—H10	119.7	C9'—C10'—H10'	119.2
C12—C11—C10	119.6 (7)	C12'—C11'—C10'	119.9 (6)
C12—C11—H11	120.2	C12'—C11'—H11'	120.1
C10—C11—H11	120.2	C10'—C11'—H11'	120.1
C11—C12—C13	121.3 (6)	C13'—C12'—C11'	119.8 (5)
C11—C12—H12	119.3	C13'—C12'—H12'	120.1
C13—C12—H12	119.3	C11'—C12'—H12'	120.1
C14—C13—C12	117.8 (7)	C12'—C13'—C14'	119.5 (6)
C14—C13—H13	121.1	C12'—C13'—H13'	120.2
C12—C13—H13	121.1	C14'—C13'—H13'	120.2
F1—C14—C13	118.1 (6)	F1'—C14'—C9'	118.4 (4)
F1—C14—C9	117.7 (4)	F1'—C14'—C13'	118.6 (5)
C13—C14—C9	124.2 (6)	C9'—C14'—C13'	123.0 (6)
			
C4—N2—C2—N1	15.5 (6)	C4'—N2'—C2'—N1'	−14.4 (6)
C4—N2—C2—S1	−164.9 (3)	C4'—N2'—C2'—S1'	166.1 (3)
C6—N1—C2—N2	3.0 (6)	C6'—N1'—C2'—N2'	−6.2 (6)
C6—N1—C2—S1	−176.6 (3)	C6'—N1'—C2'—S1'	173.3 (3)
C7—O2—C3—O1	2.2 (7)	C7'—O2'—C3'—O1'	−0.1 (7)
C7—O2—C3—C5	−176.0 (4)	C7'—O2'—C3'—C5'	178.7 (4)
C2—N2—C4—C9	99.7 (4)	C2'—N2'—C4'—C5'	26.5 (5)
C2—N2—C4—C5	−24.1 (5)	C2'—N2'—C4'—C9'	−97.8 (4)
O1—C3—C5—C6	4.1 (7)	O1'—C3'—C5'—C6'	−3.7 (7)
O2—C3—C5—C6	−177.8 (4)	O2'—C3'—C5'—C6'	177.5 (4)
O1—C3—C5—C4	−172.3 (4)	O1'—C3'—C5'—C4'	173.1 (5)
O2—C3—C5—C4	5.9 (5)	O2'—C3'—C5'—C4'	−5.6 (5)
N2—C4—C5—C6	16.0 (5)	N2'—C4'—C5'—C6'	−20.0 (5)
C9—C4—C5—C6	−106.7 (4)	C9'—C4'—C5'—C6'	103.1 (4)
N2—C4—C5—C3	−167.6 (3)	N2'—C4'—C5'—C3'	163.0 (3)
C9—C4—C5—C3	69.7 (4)	C9'—C4'—C5'—C3'	−73.8 (5)
C3—C5—C6—N1	−178.2 (4)	C3'—C5'—C6'—N1'	−179.1 (4)
C4—C5—C6—N1	−2.0 (6)	C4'—C5'—C6'—N1'	4.1 (6)
C3—C5—C6—C1	1.9 (7)	C3'—C5'—C6'—C1'	1.1 (7)
C4—C5—C6—C1	178.1 (4)	C4'—C5'—C6'—C1'	−175.7 (4)
C2—N1—C6—C5	−9.2 (6)	C2'—N1'—C6'—C5'	10.9 (6)
C2—N1—C6—C1	170.7 (4)	C2'—N1'—C6'—C1'	−169.3 (4)
C3—O2—C7—C8	164.9 (7)	C3'—O2'—C7'—C8'	−175.2 (5)
N2—C4—C9—C14	114.6 (4)	N2'—C4'—C9'—C14'	−114.4 (4)
C5—C4—C9—C14	−123.6 (4)	C5'—C4'—C9'—C14'	123.0 (4)
N2—C4—C9—C10	−63.1 (5)	N2'—C4'—C9'—C10'	65.2 (5)
C5—C4—C9—C10	58.8 (5)	C5'—C4'—C9'—C10'	−57.5 (5)
C14—C9—C10—C11	1.1 (6)	C14'—C9'—C10'—C11'	0.5 (7)
C4—C9—C10—C11	179.0 (4)	C4'—C9'—C10'—C11'	−179.1 (4)
C9—C10—C11—C12	−0.9 (8)	C9'—C10'—C11'—C12'	−1.7 (8)
C10—C11—C12—C13	1.0 (10)	C10'—C11'—C12'—C13'	0.7 (9)
C11—C12—C13—C14	−1.4 (10)	C11'—C12'—C13'—C14'	1.4 (9)
C12—C13—C14—F1	−177.6 (6)	C10'—C9'—C14'—F1'	−179.5 (4)
C12—C13—C14—C9	1.7 (9)	C4'—C9'—C14'—F1'	0.1 (7)
C10—C9—C14—F1	177.8 (4)	C10'—C9'—C14'—C13'	1.7 (7)
C4—C9—C14—F1	0.0 (6)	C4'—C9'—C14'—C13'	−178.7 (5)
C10—C9—C14—C13	−1.6 (7)	C12'—C13'—C14'—F1'	178.5 (5)
C4—C9—C14—C13	−179.3 (5)	C12'—C13'—C14'—C9'	−2.7 (9)

### Hydrogen-bond geometry (Å, º)

**Table:**

*D*—H···*A*	*D*—H	H···*A*	*D*···*A*	*D*—H···*A*
N1—H1···S1′^i^	0.88	2.83	3.703 (4)	170
N1′—H1′···S1	0.88	2.84	3.711 (4)	171
N2—H2···S1′	0.88	2.52	3.337 (4)	155
N2′—H2′···S1^ii^	0.88	2.50	3.335 (5)	158
C1′—H1′1···O1′	0.98	2.14	2.861 (6)	129

Symmetry codes: (i) *x*+1, *y*, *z*; (ii) *x*−1, *y*, *z*.
