# Supplementary figures and images for: Crystal structure of ethyl 4-(2-fluoro­phen­yl)-6-methyl-2-sulfanyl­idene-1,2,3,4-tetra­hydro­pyrimidine-5-carboxyl­ate
Source: Acta Crystallogr E Crystallogr Commun. 2015 Sep 12;71(Pt 10):o699–700. doi: 10.1107/S2056989015015145 (PMC4647406; doi:10.1107/S2056989015015145)

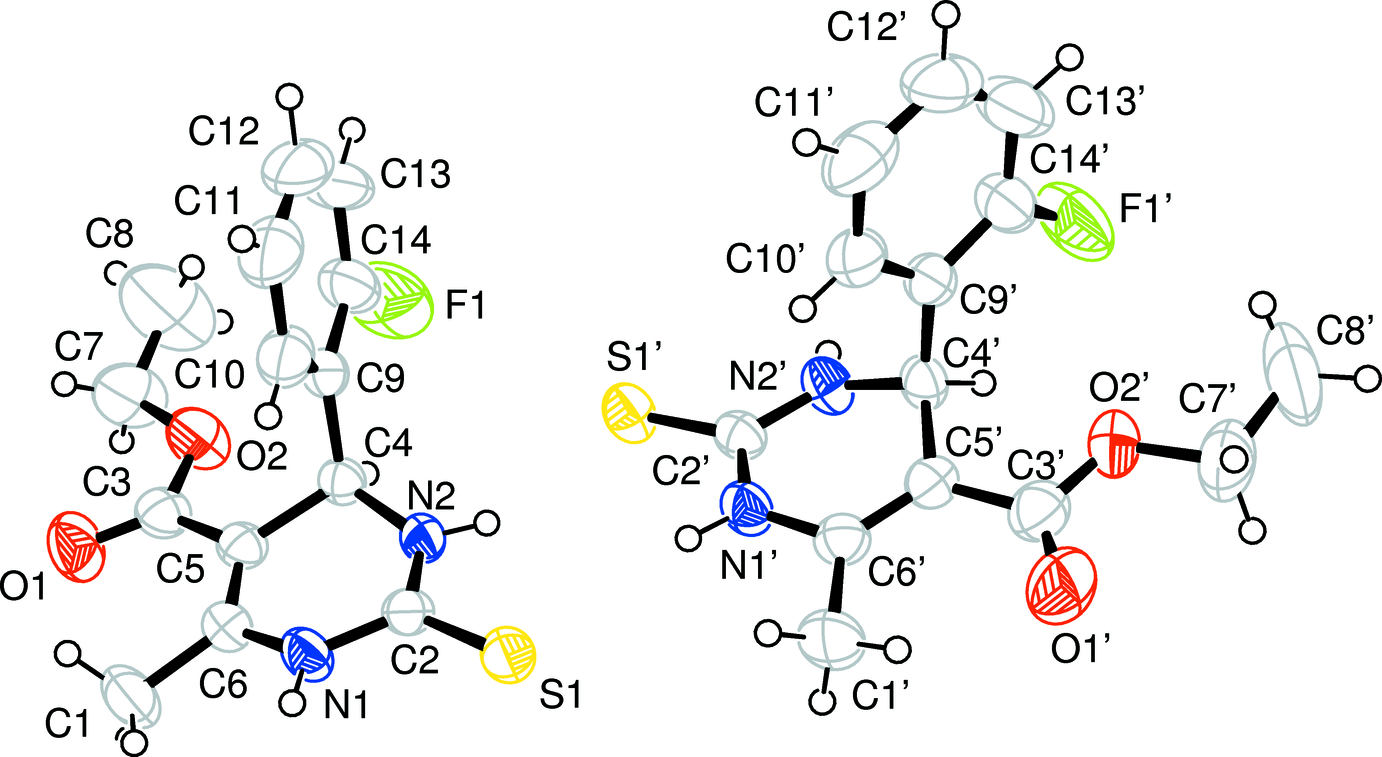

Supplement: Supplementary file 4 [file e-71-0o699-fig1.tif]

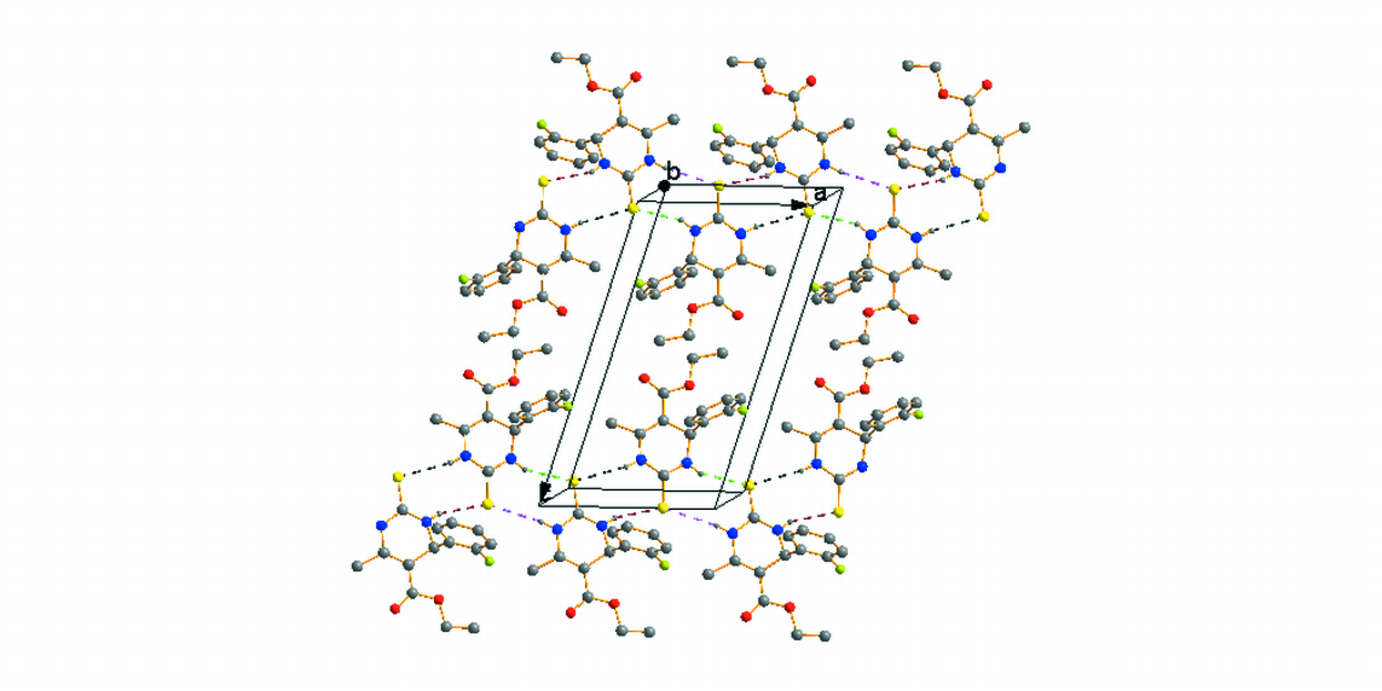

Supplement: Supplementary file 5 [file e-71-0o699-fig2.tif]
